# The latitudinal herbivory hypothesis revisited: To be part is to be whole

**DOI:** 10.1002/ece3.2759

**Published:** 2019-03-19

**Authors:** Jianguo Gao, Changming Fang, Bin Zhao

**Affiliations:** ^1^ Coastal Ecosystems Research Station of the Yangtze River Estuary Ministry of Education Key Laboratory for Biodiversity Science and Ecological Engineering Institute of Biodiversity Science Fudan University Shanghai China

**Keywords:** biotic interactions, climate, latitude, latitudinal herbivory hypothesis, macroecology, precipitation, quantile regression, temperature

## Abstract

As the big data accumulation in ecology picks up pace, we now have the opportunity to test several macroecological hypotheses, such as the latitudinal herbivory hypothesis (LHH) dated from the 1990s. The LHH proposes that plant–herbivore interactions decrease as latitude increases, that is, from lower latitudinal areas (i.e., the equator) to higher latitudinal areas (i.e., the poles). This hypothesis has been challenged in recent years. In this study, we used the greatest volume dataset of leaf herbivory from the study of Zhang et al. (*Journal of Ecology*,* 104*, 2016, 1089) to test the LHH at a global scale, based on a quantile regression model. We found that the mean annual temperature, mean annual precipitation, and potential net primary production were heterogeneously correlated with herbivory at different quantiles or variable intervals. Although the Northern Hemisphere (NH) and the global‐scale trends are in accordance with the expected latitudinal variation, the Southern Hemisphere (SH) was found to exhibit inverse trends. The latitude has a negative effect on plant–herbivore interactions in the NH and on a global scale; leaf herbivory decreased more at a given latitude in higher latitudinal areas, which is attributed to harsher survival conditions in these areas. The uniformity of leaf herbivory variability along the climate and latitude gradient in the NH and on a global scale motivates that the loosening of this herbivory variability in the SH is not significant enough to dismiss the prevalence of the LHH, a testable macroecology hypothesis.

## 1 Introduction

For years, ecologists have been curious about the mechanisms that are fundamental in maintaining biodiversity. A popular hypothesis involves the biotic interactions in tropic regions, wherein plant–herbivore interactions are stronger than in the temperate regions (Becerra, 2015; Coley & Barone, 1996; Janzen, 1970; Schemske, Mittelbach, Cornell, Sobel, & Roy, 2009). In these tropic regions, no single species can easily occupy all of the available resources, which would otherwise limit the biodiversity to one mega‐single species population (Coley & Barone, 1996; Janzen, 1970). The fossil records provide substantial evidence that the radiation of diversity in angiosperm plants and plant‐eating insects is paralleled, indicating that these two biological systems have been strongly correlated since ancient times, for example, the Cretaceous or the Pleistocene periods (Qin, 1987). Latitude‐correlated herbivory mirrored this biotic coevolution and other many important biological questions in the long geological periods (Qin, 1987).

The energy and material flows from plants to herbivores are key drivers for natural ecosystems, and herbivory is thus considered as a key component of terrestrial food webs. In the early 1990s, the latitudinal herbivory hypothesis (LHH) was proposed to explain why the herbivory and plant defense is greater in lower latitudinal areas than in higher latitudinal areas, which is the primary reason for the maintenance of a higher biodiversity in the tropics (Coley & Aide, 1991; Coley & Barone, 1996; Schemske et al., 2009). The latitudinal variation in herbivory is, in essence, proposed to be a result of the variation of water, energy, and available resource from favorable, lower latitudinal areas to harsh, higher latitudinal areas (Cox, Moore, & Ladle, 2016). For example, it is more favorable for plants and plant‐eating insects in tropical areas comparing with temperate areas, thus leading to stronger interactions between the two biological systems. The higher growth rate and production of plants provides affluent food for insects, which also means more damage to plants; this is the biological basis of the LHH. The macroecological LHH has, however, recently been questioned by several researches (cf. Adams & Zhang, 2009; Kozlov, Lanta, Zverev, & Zvereva, 2015; Moles, Bonser, Poore, Wallis, & Foley, 2011; Moles, Wallis, et al., 2011; Zhang, Zhang, & Ma, 2016), even on its reasonable ecological basis. The stronger interactions between plants and the herbivores in warmer, more humid areas where with more available resources, suggesting the variability of herbivory, were determined by local climatic factors, for example, temperature, precipitation and the productivity, or to some extent the activity of predators. Contradictory results or conclusions have arisen, owing to different studies, wherein the herbivory patterns of different plant components of the same species along a latitudinal gradient are even opposite (Anstett, Naujokaitis‐Lewis, & Johnson, 2014; Moreira, Abdala‐Roberts, Parra‐Tabla, & Mooney, 2015). In a study of the negative correlation between herbivory and temperature, the authors did not validate the LHH from the differences in the responses to environmental variables of the predators (Adams & Zhang, 2009; Björkman, Berggren, & Bylund, 2011). The intrinsic contrasting sensitivities of the plant–herbivore–predator trophic relationships to environmental variables may be responsible for published contradictory results or conclusions (Voigt et al., 2003). However, to the best of our knowledge, very few researches focused on the possible correlations between herbivory variability and different sensitivities of the trophic level from ecosystem perspective.

By taking advantage of big data accumulation in ecological aspects, ecologists now have the chance to test macroecological hypotheses (Moles et al., 2014), that is, the prevalence of the LHH, for purposes of this study. A recent meta‐analysis showed that the LHH was supported only in the Northern Hemisphere (NH), but not in the Southern Hemisphere (SH; Zhang et al., 2016), bringing this basic ecological question under scrutiny again. The same dataset from Zhang et al. (2016) was used to clarify the following questions based on a quantile regression model: (1) To what extent do the differences in slopes (the strength of plant–herbivore interactions) of leaf herbivory and climatic variables (i.e., temperature and precipitation) scale in the NH, SH, and globally? (2) Does herbivory decrease more so in higher latitudinal areas than in lower latitudinal areas for a given latitude? Answering the first question could help us to disentangle the fine herbivory variability, and revealing of herbivory variability in different climatic intervals could help explain the published contradictory results or predict plant fitness under future climatic scenarios. Owing to the unoverlooked effects of the predators or biodiversity on herbivory variability, we finally discussed the differences in sensitivity to environmental variables of the trophic level in our constructed model rather than touch the underlying mechanisms of predators to environmental variables.

## 2 Materials and Methods

### 2.1 Leaf herbivory data

We used the dataset from the study of Zhang et al. (2016), which was compiled on the basis of two former studies (cf. Lim, Fine, & Mittelbach, 2015; Turcotte, Davies, Thomsen, & Johnson, 2014; Turcotte, Thomsen, et al., 2014). The focus variable is leaf herbivory rather than flower, seed, or belowground; we focused on leaf herbivory because leaf damage is widely quantified (Andrew, Roberts, & Hill, 2012) and vital for plant fitness. Leaf herbivory is quantified using unified methods that favor the comparisons of different findings (Turcotte, Davies, et al., 2014). The leaf herbivory dataset has the largest volume so far and contains geo‐information and climatic variables. The dataset used in this study includes 166 plant families (woody and nonwoody) and more than 1000 species from 527 locations distributed worldwide (Zhang et al., 2016). The year of publication ranges from 1964 to 2014, and 1890 data points were compiled from 291 pieces of literature. Our reanalyzed dataset includes 1,297 data points from the NH and 392 data points from the SH, due to the lack of climatic variables in some research sites. A more detailed description of this dataset can be found in Zhang et al. (2016). The dataset is available at http://onlinelibrary.wiley.com/wol1/doi/10.1111/1365-2745.12588/suppinfo

### 2.2 Net primary production data

We calculated the potential net primary production (NPP) of each research site to reveal the integrated effects of temperature and precipitation on leaf herbivory based on the Miami model (Lieth, 1973). The potential NPP is the minimum of the following equations:

(1) $${\text{NPP}}_{\mathrm{MAT}}=3,000\times {\left(1+e^{1.315-0.119\;\times \;\mathrm{MAT}}\right)}^{-1}$$

(2) $${\text{NPP}}_{\mathrm{MAP}}=3,000\times \left(1-e^{-0.000664\;\times \;\mathrm{MAP}}\right)$$

(3) $$\text{NPP}=\min({\text{NPP}}_{\mathrm{MAT}},\;{\text{NPP}}_{\mathrm{MAP}})$$

where MAT and MAP denote the mean annual temperature and the mean annual precipitation, respectively.

### 2.3 Data analysis

In order to reveal the fine variability of leaf herbivory in different climatic intervals, we used a quantile regression model that is not sensitive to decentralized data points. Quantile regression is a nonparametric test that makes no assumptions regarding normality of distribution or variance homogeneity. We used the raw leaf herbivory data (%) rather than transformed data to reveal the real effects of independent variable on dependent variable, and without omitting the 0 of herbivory data. Quantile regression seeks to complement classical linear regression analysis to estimate all parts of the response distribution, that is, two environmental, NPP, and latitude variables, conditional to the predictor variable, thus providing a more comprehensive characterization of the effects than those provided by estimates of the conditional mean made with generalized least squares (GLS)/ordinary least squares (OLS) regression (Cade & Noon, 2003; Cade, Noon, & Flather, 2005; Ricotta, Godefroid, & Rocchini, 2010). Quantile regression overcomes various problems that GLS/OLS regression is confronted with. For instance, by focusing on the mean, information about the tails of a distribution is lost. By contrast, being based on absolute values rather than on squared deviations, quantile regression reduces outlier effects (Gao et al., 2016). We estimated the quantile regression functions of .05, .1, .2, .3, .4, .5, .6, .7, .8, .9, and .95 quantiles of MAT, MAP, NPP, and latitude using the R package “quantreg” (Koenker, 2013).

## 3 Results

**Figure 1:**
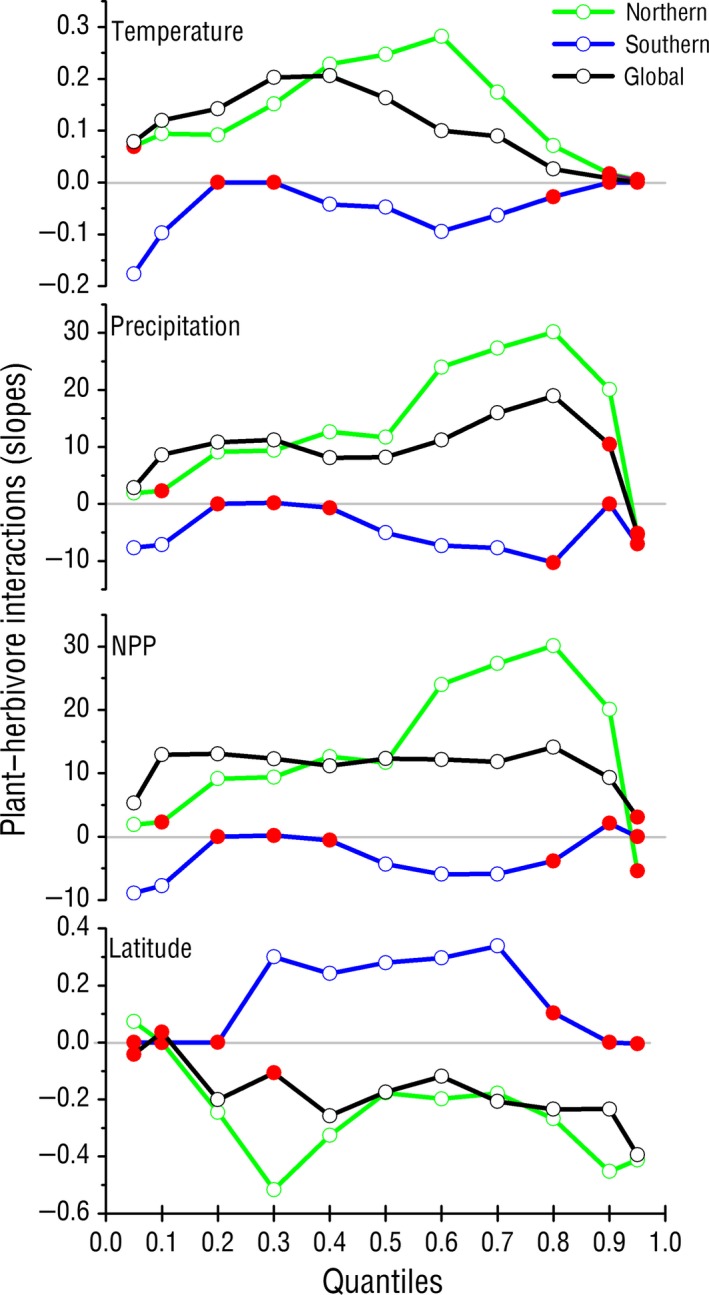
The regressed relationships between the .05, .1, .2, .3, .4, .5, .6 .7, .8, .9, and .95 quantiles of mean annual temperature, precipitation, net primary production (NPP), latitude, and leaf herbivory. The horizontal axis denotes the quantiles, and the vertical axis denotes the slopes (the strength of plant–herbivore interactions) of leaf herbivory. The green lines and circles denote the relationship in the Northern Hemisphere (NH), the blue lines and circles denote the relationship in the Southern Hemisphere (SH), the black lines and circles denote the global relationship, and the red dots denote nonsignificant relationships. This figure clearly depicts the uniformity of the latitudinal herbivory hypothesis in the NH and globally, and the limited loosening of the SH

The slopes of each quantile or the strengths of the plant–herbivore interactions are shown in Figure 1. In general, the quantiles that were lower than .3 or greater than .8 had more nonsignificant slopes (Figure 1, Table 1), especially for those in the SH. The loosening effects of the SH were mainly on the .6, .7, .8, and .9 quantiles. No matter which variable, the NH and overall globe, was highly consistent, while the SH exhibited the opposite pattern. The quantile at .6 for the MAT had the strongest effect on plant–herbivore interaction, while the .3 quantile was the strongest for the overall globe due to the attenuation of the SH. NPP and MAP had the same pattern, in which the .8 quantile showed the strongest effect.

**Table 1:** The slopes (the strength of plant–herbivore interactions) and significance of the regressed relationships between leaf herbivory (H) and mean annual temperature (MAT), mean annual precipitation (MAP), net primary production (NPP), and latitude at the .05 quantile to the .95 quantile

		Northern		Southern		Global	
		Slope	*p*	Slope	*p*	Slope	*p*
MAT~H							
τ	.05	0.06935	.21244	−0.17621	.02127	0.07846	.00403
	.1	0.09443	.00003	−0.09733	.01152	0.11977	.0000
	.2	0.09189	.00007	0.0000	1	0.1425	.0000
	.3	0.15152	.0000	0.0000	1	0.2027	.0000
	.4	0.22831	.0000	−0.04198	.00005	0.20623	.0000
	.5	0.24717	.0000	−0.04744	.00432	0.16307	.0000
	.6	0.28182	.0000	−0.09416	.0000	0.1	.00001
	.7	0.17394	.0000	−0.06299	.01872	0.08942	.0000
	.8	0.07097	.00249	−0.02731	.1382	0.02595	.00167
	.9	0.01703	.06402	0.0000	1	0.00819	.19255
	.95	0.00554	.1476	0.0000	1	0.0000	.159
MAP~H							
τ	.05	1.88375	.01773	−7.67606	.0478	2.8553	.03782
	0.1	2.31021	.26114	−7.16981	.00379	8.62919	.00376
	0.2	9.12644	.00312	0.0000	1	10.8237	.0000
	0.3	9.40147	0	0.17637	.54365	11.21718	.0000
	0.4	12.63909	0	−0.67876	.53003	8.07863	.0000
	0.5	11.69191	.00059	−5.03451	.00017	8.20046	.0021
	0.6	24.00261	0	−7.29976	.00097	11.18329	.02754
	0.7	27.31707	.00007	−7.7162	.04934	15.97444	.00791
	0.8	30.15754	0	−10.29751	.0529	18.93322	.00039
	0.9	20.1005	.04862	0.0000	1	10.46449	.10657
	0.95	−5.38793	.78732	−7.0303	.41128	−5.18336	.58058
NPP~H							
τ	.05	1.88375	.01733	−8.9052	.03943	5.28467	.01011
	.1	2.31021	.26114	−7.73617	.00368	12.9402	.00129
	.2	9.12644	.00312	0.0000	1	13.07083	.0000
	.3	9.40147	0	0.18688	.78464	12.29887	.0000
	.4	12.63909	0	−0.56368	.26621	11.17634	.0000
	.5	11.69191	.00059	−4.334	.00007	12.30503	.0000
	.6	24.00261	0	−5.91858	.00019	12.17775	.00116
	.7	27.31707	.00007	−5.88549	.03429	11.82739	.004
	.8	30.15754	0	−3.84758	.17298	14.13215	.00002
	0.9	20.1005	.04862	2.09854	.61955	9.3185	.0000
	0.95	−5.38793	.78723	0.0000	1	3.05031	.14518
Latitude~H							
τ	.05	0.07343	.0008	0.0000	1	−0.04125	.27585
	.1	−0.00062	.93432	0.0000	1	0.03629	.62005
	.2	−0.24466	.00011	0.0000	1	−0.20074	.00154
	.3	−0.51604	.0001	0.30017	.00295	−0.10659	.07706
	.4	−0.32602	0	0.24193	.00846	−0.25701	.00346
	.5	−0.1774	.00605	0.27997	.0000	−0.17431	.00084
	.6	−0.19735	.00121	0.29666	.0000	−0.11863	.00006
	.7	−0.17818	.00021	0.33842	.0000	−0.20713	.0000
	.8	−0.2674	0	0.10391	.25337	−0.23435	.0000
	.9	−0.45229	0	0.0000	1	−0.23334	.0000
	.95	−0.4119	0	−0.00391	.79404	−0.39401	.0000

*p *<* *.05 denotes significance at .05 level.

**Figure 2:**
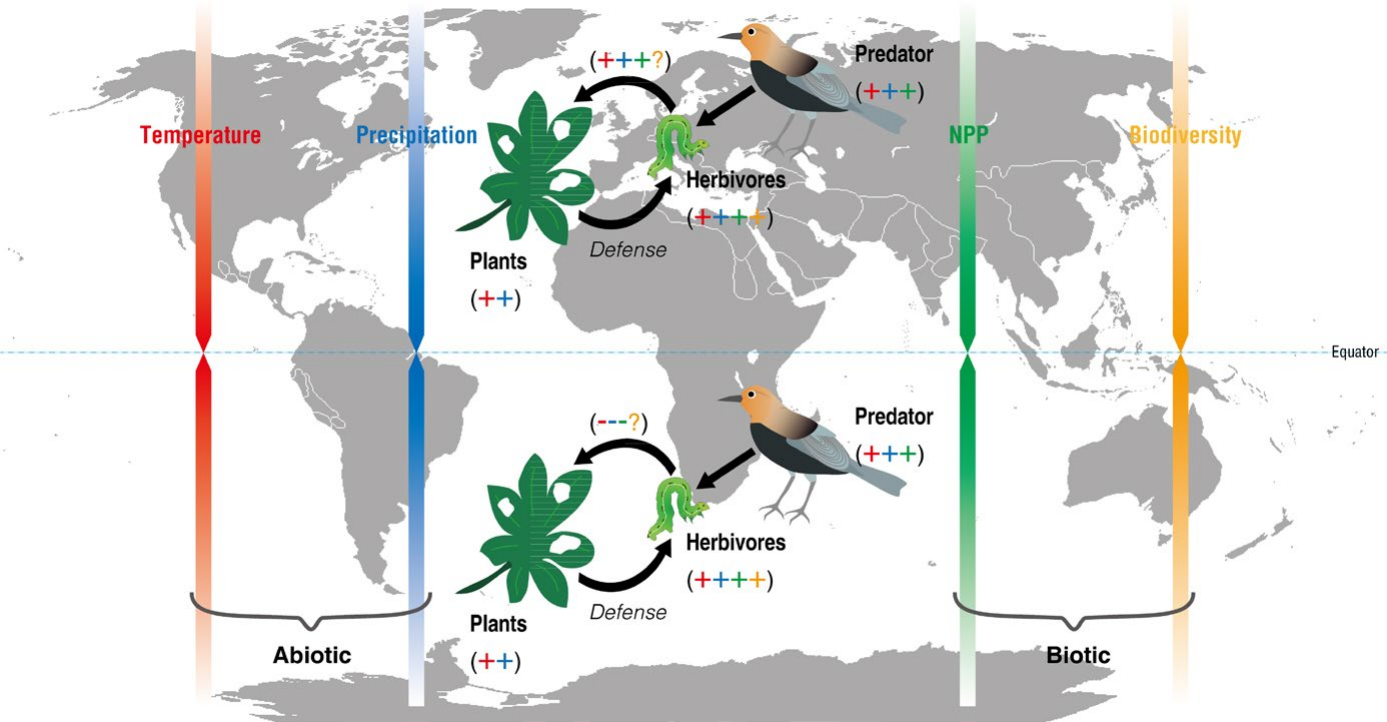
A conceptual model illustrating plant–herbivore–predator trophic relationships in the Northern Hemisphere (NH) and Southern Hemisphere (SH). The symbols “+,” “−,” and “?” denote positive, negative, and unknown effects of climatic factors, net primary production (NPP), and biodiversity on herbivory. The plants’ defenses included physical, chemical, physiological, and phonological adaptations (Coley & Barone, 1996). The red, blue, green, and orange arrows and symbols denote the variations in mean annual temperature, precipitation, NPP, and biodiversity; the darkening of the color from the poles to the equator indicates the variables are increasing (Gillman et al., 2015); and that the plant–herbivore interactions are higher in the equatorial regions than in nonequatorial areas (Becerra, 2015; Schemske et al., 2009). The contrasting leaf herbivory between the NH and the SH is probably attributed to (1) the direct effects of biodiversity on plant–herbivore interactions; (2) the differences in the sensitivity of herbivores and predators to temperature, precipitation, and NPP, which is the indirect effects on leaf herbivory. For example, even plants, herbivores, and predators have theoretically positive responses to elevated temperature, precipitation, and NPP. If the predators in the SH have an increased positive response, which would lead to higher predation pressure, the plant–herbivore interactions or the herbivory would decrease

The .3, .4, .5, .6, and .7 quantiles of latitude of the SH had a stable and significant effect on herbivory. There were stronger effects on herbivory with the higher quantiles or in higher latitudinal areas for the NH and globally; that is, the strength of plant–herbivore interactions was substantially decreased. The possible effects of temperature, precipitation, NPP, and biodiversity on the primary producers, herbivores, and predators are presented in Figure 2. We believed that, in addition to the abiotic factors—temperature and precipitation—which had direct effects on herbivory, the differences in the sensitivities of herbivores and predators to climatic variables, and the biotic factors—biodiversity and NPP—may play a more important role in the SH.

## 4 Discussion

The latitudinal variation in biotic interactions in plant–herbivore relationships, biodiversity, and NPP has long been alluring basic ecological questions, owing to the latitude representing the comprehensive effects of temperature, precipitation, and available resources (Frenne et al., 2013). The collection of data via similar methodologies in studies and comparisons is important for gaining knowledge and developing new research (Qin, 1987).

### 4.1 The latitudinal herbivory pattern and possible mechanisms

We reanalyzed the dataset of Zhang et al. (2016) using a quantile regression model to finely disentangle the variability of herbivory in different climatic, NPP, and latitudinal intervals, which confirmed that these variables have nonlinear and complicated effects on herbivory (Kim, 2014; Kozlov et al., 2015). We found that there are significant relationships at .5, .6, and .7 quantiles regardless of which biotic or abiotic variables were involved. This finding is inconsistent with those of Zhang et al. (2016), who found no significant correlation between precipitation and herbivory. The plant–herbivore interactions are found to be the strongest at the .3 quantile of MAT, that is, 9.1°C at the global scale, while the strongest interaction was found at the .6 quantile or 13.98°C in the NH. Interestingly, for higher quantiles, for example, .9 quantile of MAT (26.2°C) at the global scale, or 25.6°C, >2,000 mm of MAP, >2,000 g m^−2^ year^−1^ of NPP of those ecosystems in the NH are typical tropical forests (Whittaker, 1975), which is consistent with Kozlov et al. (2015) who reported that there is no significant relationship between temperature and woody plant foliage to insects. The plant–herbivore interactions were the strongest at the .8 quantile, that is, 1,520.3 and 1,677.9 mm of MAP for the NH and globally. These ecosystems are typical moist forests with this type of MAP. In the SH, the plant–herbivore interactions were the strongest at the .5–.7 quantile, which are typical subtropical or tropical forests with a MAP of 1219.1–1693.4 mm or NPP of 1637–2018 g m^−2^ year^−1^.

The effects of NPP on plant–herbivore interactions at the global scale are stable, and little variation was observed from the .1 to the .8 quantile, indicating that the increase in available resources does not strengthen plant–herbivore interactions, contradictory to former general knowledge (Chapin, Matson, & Vitousek, 2011; Cyr & Pace, 1993; McNaughton, Oesterheld, & Frank, 1989). We also found that the slopes decreased with latitude at the global scale, meaning that the strength of plant–herbivore interactions decreased significantly in harsh environments, which is consistent with the resource‐availability hypothesis (RAH). RAH postulated that plants invest more energy in defense in harsher environments because the cost of replacing damaged tissue is high, thus leading to low biotic interactions between plants and herbivores (Endara & Coley, 2011). Plotting of the slopes against quantiles in the latitude panel (Figure 1; Table 1) showed a significantly negative relationship at the global scale (*r* = −.77, *p *=* *.005), confirming a stronger reduction in plant–herbivore interactions in higher latitudinal areas. The plant–herbivore interactions were found to be significant at the .3–.7 quantiles in SH, that is, 8.2°–20.3° in the latitude panel, in which herbivory is negatively correlated with MAT, MAP, and NPP. We speculated that the sensitivity to temperature of natural enemy (or predator) is higher than that of the plant‐eating insects (Berggren, Björkman, Bylund, & Ayres, 2009; Björkman et al., 2011; Figure 2).

### 4.2 To be part is to be whole

The asymmetry of findings in the NH and the SH is probably attributed to the profound sampling bias that existed (Gaston, 1996) with there being almost three times more data points for the NH than for the SH; the contrasting plant functional traits between the NH and the SH as there would be more long‐leaved tropical tree species in the SH; or the potential regulation by oceanic climates in the SH.

The asymmetry of ecological patterns between the two hemispheres is not uncommon, and it has been documented in studies of the biodiversity patterns in ants (Dunn et al., 2009), spiders, New World birds (Blackburn & Gaston, 1996), and some deciduous trees (Körner & Paulsen, 2004). The most popular explanation for this asymmetrical biodiversity is that since the Eocene, there has been greater climate change in the NH than in the SH which led to more extinctions in the NH (Chown, Sinclair, Leinaas, & Gaston, 2004; Dunn et al., 2009; Mannion, Upchurch, Benson, & Goswami, 2014). The asymmetry in biodiversity or biotic interactions could profoundly affect plant–herbivore relationship (Barrio et al., 2016; Schuldt et al., 2010; Unsicker et al., 2006). The high magnitude of biodiversity in the SH probably means that there is less predation pressure on herbivores. However, it is unknown to what extent the asymmetry in biodiversity could be used to explain the leaf herbivory variability along climatic factors. The leaf biophysical traits are also considered as an important factor influencing plants’ defensive strategy; for example, Lim et al. (2015) showed that annual herbivory rates tended to be greater at lower latitudes for evergreen species (which have long‐lived leaves), but no trend in herbivory rate with latitude was found for species that had leaves with short life spans at higher latitudes. Zhang et al. (2016) reported that leaf herbivory in the SH was 1.5 times greater than that of the NH, but this difference was confirming the LHH when considering the median latitude of the SH was 16.0° (tropical regions, usually evergreen tree species) while it is 35.8° for the NH (temperate regions, usually deciduous tree species).

Another profound impact on leaf herbivory may be due to the contrasting geological or topographical features between the NH and the SH. Approximately 70% of the total land area on earth is mainly in the NH. The water:land ratio is 1:1 in the NH, while it is 16:1 between 30° and 60° latitude in SH. The ecological processes in the SH are therefore influenced more by oceanic climates than in the NH (Chown et al., 2004). In this study, we found the sampling sites in the SH were more distributed around the coastline and were closer to the equator. Even the precipitation in the SH is higher than in the NH (Zhang et al., 2016). The higher temperatures experienced in the SH probably lead to intensified drought due to more rain falling in the sea because of the less land areas, potentially intensifying plant–herbivore interactions (Coley & Barone, 1996; Lenhart, Eubanks, & Behmer, 2015).

## 5 Conclusion

The results of this study suggest that the herbivory variability in different climatic intervals should be seriously considered; that is, we found that the herbivory variability in higher quantiles of temperature and precipitation, or NPP was not significant. We also found that the loosening of the SH is not substantial, and the uniformity of the NH and global leaf herbivory variability suggests the prevalence of LHH. Although plant growth and herbivore activity should obey general biological and ecological rules (Cox et al., 2016), we speculate that other influential factors are more important than climatic factors, for example, biodiversity pattern or different intrinsic biological sensitivity of the trophic level to climatic factors. We urge that studies involving herbivory variability should therefore pay more attention to the other metrics affecting herbivory, for example, aridity level. Only through the integration of biotic interaction studies within studies involving plant defense (Moles, Wallis, et al., 2011), the degree of specialization and feeding guild (Anstett et al., 2014), plant functional traits (e.g., TRY database) (Andrew et al., 2012), and involving the abiotic climate and geology, will we collectively be able to disentangle the underlying biological mechanisms of LHH.

Several popular hypotheses have been raised from the 1960s (Connell, 1978; Janzen, 1970; MacArthur, 1972), but still a few rules remain prevalent in ecology. It is now a good time to test these hypotheses as the accumulation of big ecological data picks up pace. Some studies have reported that the classical hypothesis is not applicable in some regions or on an individual level, for example, the biodiversity asymmetry of Western Hemisphere and Eastern Hemisphere or the herbivory asymmetry in the NH and the SH. However, if we focus on the “whole” of the macroecological theory, the nonconsistency in “part” will not impact our understanding of the basic ecological mechanisms. The macroecological theory is still a powerful tool in predicting ecosystem structure and function under changing climate scenarios in the absence of detailed ecological process of all ecosystems.

## Conflict of Interest

Authors claim no conflict of interest.
